# Attractiveness of medical disciplines amongst Swiss first-year medical students allocated to different medical education tracks: cross-sectional study

**DOI:** 10.1186/s12909-022-03313-x

**Published:** 2022-04-07

**Authors:** Stefan Markun, Ryan Tandjung, Thomas Rosemann, Nathalie Scherz, Oliver Senn

**Affiliations:** 1 Institute of Primary Care, University and University Hospital Zurich, Zurich, Switzerland; 2 Tertianum Management CH, Dübendorf, Switzerland; 3 Division of Internal Medicine, Centre Hospitalier de Bienne, Bienne, Switzerland

**Keywords:** Medical school, Medical students, Career choice, Medical disciplines

## Abstract

**Background:**

As most countries, Switzerland is experiencing a shortage of physicians especially in general practice and new medical education tracks with respective focusses have been started in response. This study investigated Swiss medical students’ career openness and attractiveness of different medical disciplines as well as the concordance of students’ career intentions with assigned medical education tracks.

**Methods:**

Cross-sectional study surveying first year medical students assigned to four different Swiss medical education tracks with distinctive additional education focuses (ETH Zurich: medical technology and engineering, University of St. Gallen and University of Lucerne: primary healthcare and University of Zurich: no distinctive focus).

**Results:**

We surveyed 354 medical students (response rate across all included medical education tracks 71.1%), 64.8% female, mean age 20 years. Regarding career openness, we found that 52.8% of medical students had neither a strong commitment nor a strong reservation for any of the proposed career options and 17.0% had a strong commitment. Among medical disciplines, medical subspecialties were attractive to the largest part of students (inpatient subspecialties attractive for 71%, outpatient for 58%), attractiveness of general practice was moderate (30%), academic (22%) and industrial sector (17%) careers were least attractive. Proportions of medical students attracted to general practice were similar at medical education tracks with focus on primary healthcare compared to other medical education tracks (32.2% vs. 25.8%, *p* = 0.391). Conversely, proportions of medical students attracted to academic or industry careers were significantly higher at the ETH Zurich compared to other medical education tracks (37.2%, vs. 13.1%, *p* < 0.001 and 31.9%, vs. 8.8%, *p* < 0.001 respectively).

**Conclusion:**

While most first-year medical students were open to careers in many medical disciplines, attractiveness of disciplines varied strongly. Students attracted to academic or industrial careers accumulated at the medical education track with concordant teaching focus but students attracted to general practice did not accumulate at medical education tracks focused on primary healthcare. For medical education tracks with primary care teaching focus this is both a challenge and an opportunity to specifically counteract the shortage of general practitioners in Switzerland.

**Supplementary Information:**

The online version contains supplementary material available at 10.1186/s12909-022-03313-x.

## Background

Healthcare systems require a sufficiently sized and appropriately specialized medical workforce meeting population demands of medical services. In Switzerland, the number of graduates from medical school is insufficient to meet national demands and the Swiss healthcare system currently depends on a third of physicians educated abroad [1]. The supply with general practitioners is of special concern in this context because of the central role of primary care in the healthcare system and current shortages were predicted to increase in the next decade [2, 3]. To counteract the shortage in medical workforce, the Swiss Federal Council introduced financial incentives for Swiss universities to increase capacity for medical education (“Sonderprogramm Humanmedizin”) [4]. In response, several universities applied and were granted to start new medical education tracks which are composed of fixed and coordinated bachelor’s and master’s programs each comprising 3 years of medical education and both being required to obtain a medical diploma in Switzerland.

Among the newly founded medical education tracks, many feature a distinctive additional education focus. Specifically, among the newly funded medical education tracks are: Master’s programs at the universities of Lucerne and St. Gallen with additional focus on primary healthcare (the corresponding bachelor’s programs are nested in the already existing medical education track at the University of Zurich which has no additional focus); and a bachelor’s program at the ETH Zurich with and additional focus on medical technology and public health [5–7]. Since applications to all Swiss medical education tracks combined far outnumber their capacities, the number of new graduates will not be the main limitation for proliferation of the Swiss medical workforce. To fill the gaps in the future medical workforce, however, also the actually pursued careers will have to meet population demands especially in general practice. Given the specific education focusses of the newly funded medical education tracks, it is reasonable to assume that individual tracks will contribute differently to the future medical workforce. This, especially with respect to primary care because experiences during medical education are known to impact according career choices [8–11]. Foreseeably, the newly funded medical education tracks with focus on primary healthcare will be evaluated for their contribution to the general practice workforce. For such evaluations, however, it is important to know the starting conditions of career intentions of students allocated to these different medical education tracks including comparisons to medical education tracks with different educational focuses.

While it seems reasonable to assume a certain concordance between medical students’ career goals and medical education tracks’ education focusses, there is no data supporting this assumption and also there is a significant factor in play that could disturb this concordance, namely, the intricate admission process to Swiss medical education tracks. Currently, access to most Swiss medical education tracks is regulated by a national competitive aptitude test “Eignungstest” (ET) to preselect applicants. The Swiss ET is a standardized cognitive written test applied during a same day morning and an afternoon session. The test was originally developed in Germany, features several cognitive domains important for studying medicine and has been shown to be highly correlated with successful completion of medical school in Germany [12] and with academic performance during the first year of medical school in Switzerland [13]. In 2019, the number of applicants to the ET exceeded available study places by a factor of three, accordingly, two thirds of applicants were rejected [14]. The potential discordance of career goals and medical education tracks’ teaching focuses is introduced after the preselection and is due to a necessary re-distribution process managing disparities between applications to specific medical education tracks and actually available study places therein. This re-distribution process considers first the domicile (applicants living in the canton of the respective medical education tracks are favored) and secondly the score achieved in the ET (higher ranks are favored) [15]. In consequence, in 2019, at medical education tracks with focus on primary healthcare, the re-distribution assigned more than half of the students discordantly to their original application and naturally, this introduced a significant potential of discordance between students’ career goals and education focus (see supplementary file 1 for assignment flow chart, underlying data are courtesy of swissuniversities, note that the full Swiss application and allocation flow is displayed and that this study is reporting data from a subgroup of medical education tracks only). Interestingly, at the ETH Zurich with a technology and research-oriented focus the re-distribution process caused almost no re-assignments meaning that a considerably smaller discordance between career goals and education focus can be expected.

Given that new medical education tracks were funded in the intention to fill the gaps in the future medical workforce especially in general practice, there is a need for monitoring career intentions and career adoption throughout the education process. In this study, we pursued to determine the starting conditions regarding career intentions and relevant determinants thereof in first-year medical students allocated to specific medical education tracks. Specifically, we aimed to (1) assess career openness and the attractiveness of careers in different medical disciplines, (2) to explore the importance of determinants of career choice, (3) explore concordance of medical students’ career goals and assigned medical education tracks’ distinctive education focuses (considering also the potential impact of the re-distribution process) and (4) to explore associations of student characteristics with medical discipline attractiveness.

## Methods

### Design and participants

We performed a cross-sectional study among all 2019 first-year Swiss medical students assigned to the medical education tracks at the Swiss Federal Institute of Technology (ETH Zurich track), University of Zurich (UZH track), the university of Lucerne (Lucerne track) and the university St. Gallen (St. Gallen track). While the ETH Zurich track is separate starting from the bachelor’s program, the other three tracks share most courses of the bachelor’s program located at the university of Zurich to be most noticeably divided in the respective tracks when entering the master’s program [16]. Students were invited to complete the survey during a lecture in the second week after entering medical education tracks and given dedicated time for completion. The survey was in German language and available online using the survey platform Surveymonkey®. Other students present in lectures (e.g. dentistry or veterinary medicine) were excluded. Participation at the survey was voluntary and data was collected anonymously. Participants were informed that data collected in this survey will be synthesized and published. This survey did not require ethical committee approval because it did not fall under the scope of the Swiss Federal Act on Research involving Human Beings [17]. Methods were performed in accordance with the relevant guidelines and regulations.

### Questionnaire

The questionnaire was in German language, self-administered and specifically designed for the study purposes. The questionnaire consisted in eight items organized in three parts. Part one determined the medical education track the student originally applied to and the actually assigned medical education track. Part two determined the attractiveness of careers in different medical disciplines and importance of determinants of career choice both on a five-point Likert scales (ranging from “excluded career goal” to “the only career goal” and from “very unimportant” to “very important” respectively). Medical disciplines presented were: general practice, gynecology / pediatrics, outpatient subspecialty, inpatient internist, inpatient subspecialty, industrial sector, academic career. Medical disciplines were selected and grouped based on appraisal of similar previous studies investigating medical student career choice and adapted as it pertains to the Swiss medical workforce context [18–20]. Determinants of career choice presented were: financial success, reputation, political environment, part time work, relation to patients, medical tasks, career opportunities, autonomy. Determinants were selected and grouped based on appraisal of similar previous studies investigating importance of factors for career choice and adapted to the Swiss context [18, 19, 21–24]. In contrast to previous studies we eliminated student loans from the list of potential determinants because tuition is largely funded publically in Switzerland but we included the political environment because it importantly regulates working opportunities after medical education (especially favoring outpatient primary care services such as general practice over medical subspecialties). Part three determined demographic information and the ET score. The questionnaire has been piloted with 262 first-year medical students from the University of Zurich in 2018 and improved accordingly (the questionnaire has been translated in English and is available as supplementary file 2).

### Statistical analysis

Overall career openness was defined as follows: “completely open” were respondents neither rating any of the of the career attractiveness items as “the only career goal” or as an “excluded career goal”; “committed” were respondents who rated any career as “the only career goal” and “partially open” were remaining respondents (only excluding specific career goals without committing to any). Attractiveness of careers in different medical disciplines and importance of different determinants of career choice we report graphically and using counts and proportions gathering the ratings “rather attractive” or “the only goal” together into an “attractive” category. Accordingly, we considered determinants of career as being perceived to be “important” if the ratings “rather important” or “very important” were given. Analyses were stratified by medical education tracks and by medical education tracks’ distinctive teaching focuses (primary healthcare focus vs. no primary healthcare focus and technology focus vs no technology focus). We performed group comparisons between medical education tracks and within medical education tracks by differentiating students assigned to the respective medical education track concordantly to their own application vs. those re-assigned. For group comparisons, were used the Chi-squared test, Fisher’s exact test, ANOVA or Kruskal Wallis test as appropriate. Associations with attractiveness of careers, we explored using bivariate correlations by transforming the five-point Likert scale items to numerical values (− 2, − 1, 0, 1, 2) and using Spearman’s R and Spearman test (bivariate results shown in Supplementary files only). We further assessed predictors of career attractiveness by linear regression models using numerically transformed Likert scale ratings of career attractiveness as dependent variables and as independents variables students’ sex, age, ET percentage score and the likewise numerically transformed importance of determinants of career choice ratings. A two-sided *p*-value of < 0.001 was used to assert statistical significance to accommodate for multiple testing in this explorative study. Statistical analysis was performed using R version 3.6.2.

## Results

### Sample characteristics

In 2019, 498 medical students were allocated to the medical education tracks included in this study (see supplementary file 1) and thereof 354 students participated in the survey (overall response rate 71.1%). Respondents were 64.8% female, aged 20.2 (SD = 2.2) years on average and 54.7% had domicile in the canton of Zurich when they filed their application to medical education tracks (see Table 1 section 1 for characteristics of medical students stratified by medical education track).

**Table 1:** Characteristics of medical students stratified by medical education tracks

	ETH Zurich track (*n* = 94)	UZH track (*n* = 201)	Lucerne track (*n* = 32)	St. Gallen track (*n* = 27)	p*
**Section 1) Demographical characteristics**					
sex = female (%)	55 (61.1)	105 (63.3)	23 (82.1)	16 (69.6)	0.201*
age (mean (SD))	19.9 (1.8)	20.2 (2.2)	21.5 (3.8)	19.9 (1.0)	0.01^†^
assignment concordant with application (%)	76 (80.9)	199 (99.5)	14 (45.2)	16 (59.3)	< 0.001*
ET (median [IQR])	91.5 [84.0, 96.0]	89.0 [79.0, 96.0]	85.0 [78.0, 90.0]	89.5 [81.2, 91.8]	0.031^‡^
Domicile in medical education track canton (%)	34 (36.2)	132 (65.7)	6 (18.8)	12 (44.4)	< 0.001*
**Section 2) Overall career openness (%)**					
completely open	51 (54.8)	96 (56.1)	10 (32.3)	11 (47.8)	< 0.001*
partially open	17 (18.3)	52 (30.4)	18 (58.1)	9 (39.1)	
committed	25 (26.9)	23 (13.5)	3 (9.7)	3 (13.0)	
**Section 3) Number of students rating specific career as at least rather attractive (%)**					
general practice	25 (26.6)	51 (25.4)	11 (34.4)	8 (29.6)	0.740*
outpatient care: gynecologist/pediatrics	26 (27.7)	44 (21.9)	10 (31.2)	7 (25.9)	0.560*
outpatient care: subspecialty	62 (66.0)	95 (47.3)	16 (50.0)	12 (44.4)	0.020*
inpatient care: general internist	44 (46.8)	78 (38.8)	14 (43.8)	16 (59.3)	0.178*
inpatient care: subspecialty	71 (75.5)	120 (59.7)	18 (56.2)	17 (63.0)	0.049*
research & education (academic career)	35 (37.2)	30 (14.9)	1 (3.1)	3 (11.1)	< 0.001*
research & development (industry)	30 (31.9)	19 (9.5)	1 (3.1)	3 (11.1)	< 0.001*
**Section 4) Number of students rating specific determinants as at least rather important (%)**					
financial success	31 (33.0)	81 (40.3)	17 (53.1)	13 (48.1)	0.174*
reputation	40 (42.6)	70 (34.8)	11 (34.4)	12 (44.4)	0.508*
political environment	24 (25.5)	35 (17.4)	3 (9.4)	3 (11.1)	0.111*
part time working	45 (47.9)	77 (38.3)	15 (46.9)	11 (40.7)	0.426*
relationship to patients	71 (75.5)	136 (67.7)	25 (78.1)	20 (74.1)	0.400*
having medical tasks	72 (76.6)	144 (71.6)	25 (78.1)	21 (77.8)	0.707*
career opportunities	76 (80.9)	134 (66.7)	18 (56.2)	20 (74.1)	0.024*
autonomy	62 (66.0)	102 (50.7)	20 (62.5)	13 (48.1)	0.065*

^*^statistical significance of between-medical education track differences is determined by Chi-squared test(*)

^†^ANOVA

^‡^Kruskal Wallis test

Overall, 86.6% of assignments to medical education tracks matched the students’ first choice in their original applications but this ratio varied strongly between medical education tracks (lowest: 45.2% Lucerne track and highest: 99.5% UZH track).

### Career openness and attractiveness of medical disciplines

**Fig. 1:**
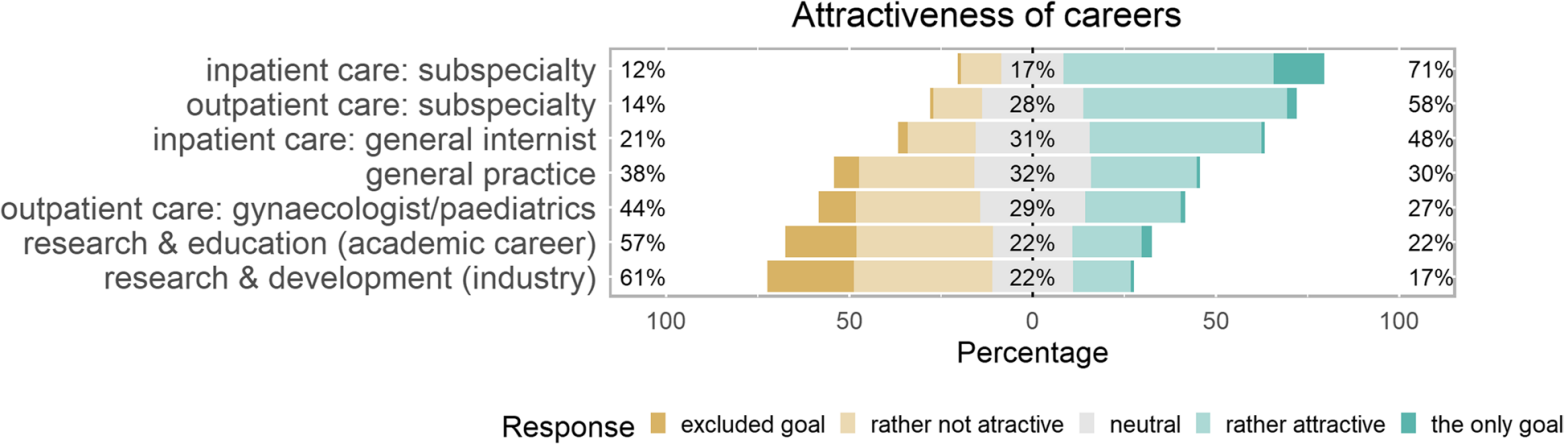
Attractiveness of career options as horizontal stacked bars centered according to distribution of responses. Bars tending to the right represent a predominance of “attractive” ratings, bars tending to the left a predominance on “not attractive” ratings. Colors represent the proportions of actual ratings

We found 52.8% of medical students completely open towards different careers, 30.2% partially open (only excluding specific careers) and 17.0% were already committed to specific disciplines (see Table 1 section 2 for stratification of openness by medical education track). Attractiveness of career options varied substantially (Fig. 1). Subspecialties received highest ratings (inpatient subspecialties attractive for 71% of medical students and outpatient subspecialties for 58%). Less than half as many students rated general practice or outpatient care gynaecology/paediatrics to be attractive (30 and 27% respectively). Academic career and career in the industrial sector were attractive to least students (22 and 17% respectively). Attractiveness ratings of career options were distributed similarly among medical education tracks (Table 1 section 3) with the exception of academic career and career in the industrial sector which were attractive for more than twice as many students at ETH Zurich track compared to the other tracks.

### Importance of determinants of career choice

**Fig. 2:**
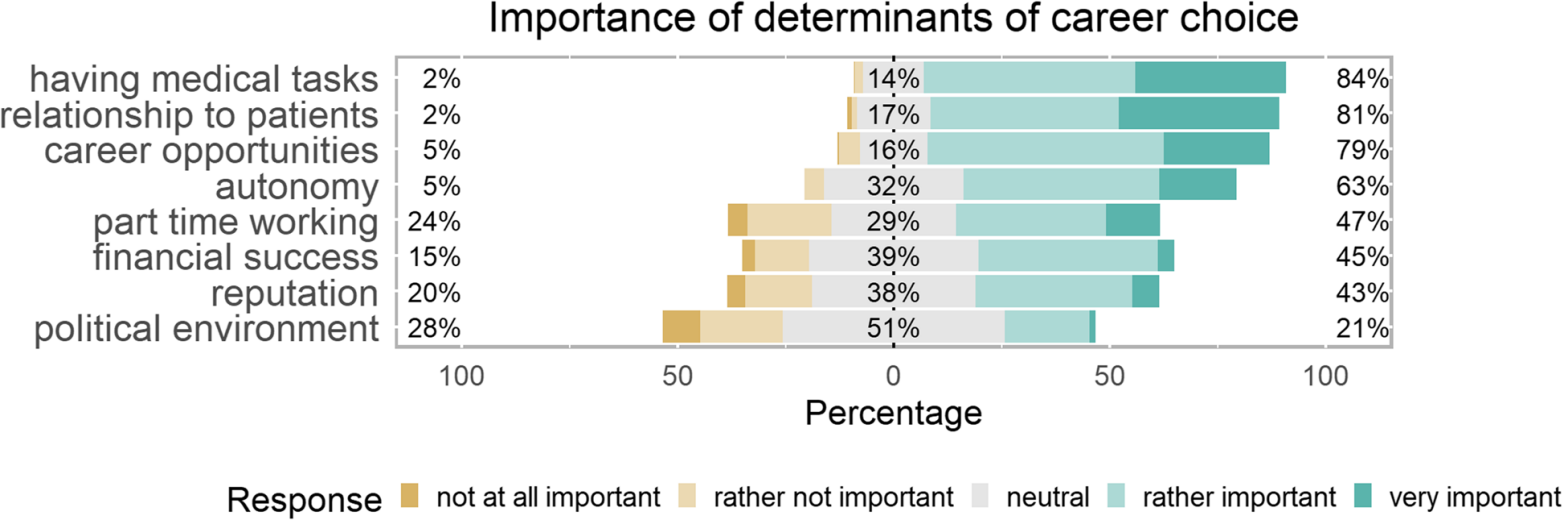
Importance of determinants of career choice as horizontal stacked bars centered according to distribution of responses. Bars tending to the right represent a predominance of “important” ratings, bars tending to the left a predominance on “not important” ratings. Colors represent the proportions of actual ratings

Importance of determinants of career choice varied strongly (Fig. 2). Determinants rated to be important by most medical students were having medical tasks (84%), relationships to patients (81%), career opportunities (79%) and autonomy (63%). Less than half of students rated part time working (47%), financial success (45%) and reputation (43%) as important. The political environment (21%) was important for a small minority of students. Importance ratings of determinants of career choice were distributed similarly among medicals education tracks (Table 1 section 4).

### Concordance of medical discipline attractiveness and education tracks’ teaching focus

The proportion of medical students attracted to a career in general practice was similar at medical education track with and without distinctive focus on primary healthcare (32.2% vs. 25.8%, *p* = 0.391). Subgroup analyses within education tracks with distinctive focus on primary healthcare (St. Gallen and Lucerne) showed that the attractiveness of a career in general practice was similar among the (*n* = 30) students assigned to these medical education tracks in concordance with their application compared to the (*n* = 28) students re-assigned (32.1% vs. 33.3% *p* = 1.0).

The proportion of medical students attracted to an academic career or a career in the industrial sector was significantly higher at the ETH Zurich track having a distinctive focus on medical technology and public health compared to other medical education tracks (academic career 37.2%, vs. 13.1%, *p* < 0.001; career in the industrial sector 31.9%, vs. 8.8%, *p* < 0.001). Subgroup analyses within students at the ETH Zurich showed that the attractiveness of academic career was similar among the (*n* = 76) students assigned to the ETH Zurich by their own first choice compared to the (*n* = 18) students re-assigned (38.2% vs. 33.3% *p* = 0.913) but the attractiveness of a career in the industrial sector differed considerably although not statistically significantly (36.8% vs. 11.1%, *p* = 0.068).

### Associations of student characteristics with subspecialty attractiveness

Importance of determinants of career choice were associated with attractiveness ratings of several disciplines. The attractiveness of general practice was associated with perceiving relationships to patients and autonomy as important and not perceiving as important career opportunities and political environment. The attractiveness of inpatient subspecialties, academic career and career in the industrial sector were all associated with perceiving career opportunities as important (see supplementary file 3 for all according bivariate associations). Linear regression predicting attractiveness of careers (five-point Likert scales transformed to numeric values − 2,1,0,1,2) using demographic factors, ET score and importance ratings of career choice as determinants (also as numerically transformed Likert items) revealed associations with demographic factors: Female sex compared to male was positively associated with attractiveness of gynecologist / pediatrician (estimate = + 0.6 points on 5-point Likert scale; *p* < 0.001) and negatively with academic career (estimate = − 0.4 points, *p* = 0.011) and career in the industrial sector (estimate = − 0.5 points, *p* = < 0.001). Age was negatively associated with attractiveness of general practice (estimate = − 0.1 points on 5-point Likert scale per year of age, p = < 0.001). Remarkably, the ET rank was not significantly associated with attractiveness of any career (see Table 2 for detailed results of linear regression models).

**Table 2:** Linear models predicting attractiveness ratings of different medical specialties

	**general practice**			**gynecologist/pediatrics**			**outpatient care: subspecialty**			**inpatient care: general internist**		
	Estimate	Std. Error	*p*	Estimate	Std. Error	*p*	Estimate	Std. Error	*P*	Estimate	Std. Error	*p*
Intercept	1.98	0.76	0.010	−0.96	0.80	0.229	−0.50	0.69	0.469	0.51	0.76	0.497
ET score	0.00	0.01	0.773	0.01	0.01	0.202	0.00	0.01	0.631	0.00	0.01	0.720
sex female	0.00	0.11	0.983	0.63	0.12	< 0.001	−0.08	0.10	0.430	0.02	0.11	0.851
age	−0.09	0.02	< 0.001	−0.02	0.02	0.340	0.03	0.02	0.127	−0.03	0.02	0.263
financial success	−0.08	0.07	0.258	−0.08	0.07	0.249	0.14	0.06	0.026	0.01	0.07	0.831
career opportunities	−0.41	0.08	< 0.001	−0.08	0.08	0.332	0.09	0.07	0.195	−0.02	0.08	0.797
autonomy	0.16	0.07	0.029	−0.05	0.08	0.520	0.01	0.07	0.852	0.04	0.07	0.613
having medical tasks	−0.15	0.08	0.060	−0.02	0.08	0.818	−0.04	0.07	0.575	0.15	0.08	0.054
part time working	0.12	0.05	0.030	0.18	0.06	< 0.001	0.04	0.05	0.465	0.12	0.05	0.023
political environment	−0.22	0.06	< 0.001	− 0.01	0.07	0.910	0.05	0.06	0.371	−0.03	0.06	0.598
relationship to patients	0.20	0.07	0.003	0.14	0.07	0.043	0.01	0.06	0.876	−0.12	0.07	0.075
reputation	0.12	0.07	0.072	0.00	0.07	0.978	−0.03	0.06	0.575	−0.08	0.07	0.218
	**inpatient care: subspecialty**			**academic**			**industry**					
	Estimate	Std. Error	*p*	Estimate	Std. Error	*p*	Estimate	Std. Error	*p*			
Intercept	0.46	0.71	0.523	−1.59	0.93	0.087	0.07	0.86	0.937			
ET score^a^	0.00	0.01	0.769	0.01	0.01	0.067	0.00	0.01	0.600			
sex female	−0.18	0.11	0.099	−0.36	0.14	0.011	−0.47	0.13	< 0.001			
age	0.01	0.02	0.614	0.01	0.03	0.806	−0.03	0.03	0.235			
financial success	0.03	0.06	0.658	−0.08	0.08	0.348	0.07	0.08	0.346			
career opportunities	0.29	0.07	< 0.001	0.37	0.10	< 0.001	0.27	0.09	0.003			
autonomy	−0.01	0.07	0.927	0.10	0.09	0.233	0.16	0.08	0.053			
having medical tasks	0.17	0.07	0.018	−0.32	0.10	< 0.001	− 0.30	0.09	< 0.001			
part time working	−0.12	0.05	0.015	0.09	0.06	0.178	0.03	0.06	0.665			
political environment	0.00	0.06	0.959	0.10	0.08	0.205	0.10	0.07	0.142			
relationship to patients	−0.13	0.06	0.035	0.01	0.08	0.937	−0.11	0.08	0.144			
reputation	−0.04	0.06	0.523	−0.06	0.08	0.425	−0.05	0.08	0.484			

^a^ET score: The percentage rank achieved in the competitive entrance examination (“Eignungstest”). Higher scores represent better ranking

## Discussion

This survey showed that only one of six medical students were committed to a specific career at the beginning of Swiss medical education tracks and most of the students were still completely open to all the proposed career options. Careers in medical subspecialties appealed to two thirds of medical students, careers in general practice to one third and academic or industry careers to a fifth. At the ETH Zurich track featuring an education focus on medical technology and public health, there were twice as many students attracted to scientific or industry careers compared to other medical education tracks. In contrast, proportions of students attracted to a career in general practice were not significantly higher at medical education tracks with distinctive focus on primary healthcare. In addition, concordance of assignment and application to specific medical education tracks was not importantly associated with career attractiveness. Attractiveness of careers was associated with several factors including importance ratings of determinants of career choice but also demographic characteristics.

The majority of medical students was open towards most of the careers proposed in this survey. This finding is in concordance with previous studies from US and Canada showing broad career openness among medical students [21, 25, 26]. Medical subspecialties being more attractive compared to careers in general practice has also been shown in previous Swiss and international studies [19, 27, 28]. In the longitudinal perspective, studies show that attractiveness of general practice increases during medical education, potentially because of positive experiences [29, 30]. The proportion we found of 30% of medical students perceiving general practice as an attractive career could be considered as high for Switzerland, especially when considering results from surveys among medical students from 20 years ago where only about 10% of medical students aspired a career in general practice [28]. When comparing internationally, the attractiveness of general practice in Swiss medical students still ranks below ratings from UK medical students where 30 to 40% of medical students are inclined towards a career in general practice [31–33].

We found that the ETH Zurich harbored significantly more medical students attracted to careers concordant with its distinctive teaching focus compared to other medical education tracks. A strong association of specific career goals with a specific medical school is rather unexpected in the light of previous research especially in first-year medical students [34]. For this successful matching, several reasons are possible such as the pertinent reputation of the ETH Zurich or a performance advantage of basic science-oriented students in the ET itself, granting them first priority to obtain study places at the medical education track they wish. Indeed, students admitted to the ETH had the highest ET scores on average not only adding to the credibility of such successful self-matching but also arguing for the accuracy of self-reported ET scores in this study. Conversely, medical students assigned to the medical education tracks at Lucerne and St. Gallen reported lower ET scores. While this result is consistent with the comparably high rates of re-assigned medical students at these medical education tracks, it also raises the concern that these students may show lower academic performance (as predicted by the ET) and thus suffer from higher drop-out rates from medical school. The numerical range of average ET scores by medical education tracks, however, was rather small, statistical significance was marginal (range from 85.0 at Lucerne to 91.5 at ETH, *p* = 0.03) and whether this difference will translate relevant difference in drop-out rates must still be observed.

With regard to the political agenda of increasing the domestic medical workforce in Switzerland, it can be criticized that basic medical science and public health oriented medical education may be less likely to contribute to the future medical workforce because formal public health education has been shown to be associated with non-clinical career outcomes of medical school graduates [35]. In this regard, however, three aspects must be acknowledged: First, the large majority of medical students at the ETH Zurich are attracted to clinical disciplines. Second clinical disciplines are still the mainstay of the education program at the ETH Zurch. Third, the COVID-19 pandemic has revealed the need for higher investments and preparedness in the public health sector and better use of digital health technologies [36, 37]. Therefore, education in public health and health technologies –while potentially being at the expense of the clinical workforce– may still very much be in the public interest and compatible with updated political agendas.

Interestingly and in contrast to the ETH Zurich, no increased concordance between career attractiveness and medical education tracks’ additional teaching focus was found in medical education tracks focusing on primary healthcare. In addition, according to subgroup analyses, even though every second medical student was re-assigned to one of these medical education tracks, the re-assignment was apparently not responsible for the observed concordance gap. For this observation one explanation could be that these medical education tracks were just newly founded, had no chance to acquire a pertinent reputation and the transition to the masters’ programs with focus on primary healthcare was still in the future when the survey took place. Moreover, there is evidence that attractiveness of general practice tends to increase during undergraduate medical education [29]. This finding of medical students assigned to the medical education tracks at Lucerne and St. Gallen not being particularly interested in a career in general practice, emphasizes the potential of these medical education tracks to actually contribute to the Swiss general practice workforce. This, because a GP-orientated undergraduate medical has indeed to potential to raise medical schools’ output of future GPs [8–10]. Also future evaluations of these medical education tracks should not wrongly assume favorable starting conditions in this respect.

We found that relationships to patients and autonomy were comparatively important to students attracted to general practice which is in line with similar Swiss and international studies [19, 31]. Also, in line with results from previous studies, we found a positive association of female sex with attractiveness gynecology/pediatrics [27, 38]. In addition, we investigated the association with career in academic or industry sectors and found a strong negative association with female sex. This finding suggests that the existing gender differences in academic medicine may already be present in medical students’ mindsets at the very beginning of their careers and fostering gender diversity may require interventions early in medical school [39].

This study has the following strengths and limitations: The high response rate argues for representativeness of the survey. Also, this is the first survey allowing direct comparisons between Swiss medical education tracks regarding important aspects of career development for inscribed students. Moreover, our results are relevant because career intentions at entry to medical school were found to predict specialty choice [24, 40]. Limitations to this study are linked to its exploratory nature and thus, interpretation of results needs careful consideration: First, small subgroups in specific analyses as for example in smaller medical education tracks such as the St. Gallen track (*n* = 27) are associated with limited power to detect between-group differences. Second, there is a risk of false-positive findings because we performed multiple tests in our data analysis. However, we set the level of statistical significance to *p* < 0.001 to minimize the risk of such alpha errors. Moreover, information from non-respondents was unavailable and because the survey was distributed to those students present at the universities, there is a risk for selection bias linked to students’ willingness to attend lectures. Lastly, career commitment in the majority of Swiss physicians happens during residency, therefore results from this studies are projections likely associated with career outcomes, not career outcomes themselves [41, 42].

## Conclusion

Medical students were open to many different careers at the beginning of medical education tracks. Students attracted to academic or industry careers seemed to self-select to study at the ETH Zurich track featuring a concordant teaching focus. Medical students assigned to medical education tracks in Lucerne and St. Gallen (with teaching focus on primary healthcare) were not more attracted to general practice compared to students at other medical education tracks. The student re-assignment process considering the score at the ET did not seem to majorly interact with concordance of students’ career goals and medical education tracks’ teaching focus. The concordance gap between career attractiveness in general practice and medical education teaching focus is both a challenge and an opportunity to meet the political goals of increasing the general practice workforce in Swizerland.

## Supplementary Information

**Additional file 1: Supplementary file 1.** Sankey plot showing proportions and (re-)assignment flow of applicants to specific medical education tracks in 2019. The first vertical bar from the left represents the total of applicants, the stack of vertical bars represents the proportion of applicants to specific medical education tracks, the third stack of bars the proportion of applicants passing the aptitude test (“Eignungstest”) and the forth stack of bars the actual allocation to specific medical education tracks. At each node the total number applicants is given.

**Additional file 2: Supplementary file 2.** Questionnaire translated in English.

**Additional file 3: Supplementary file 3.** Scatterplot matrix for all pairs of attractiveness of career goals and importance of factors associated with career. Spearman correlation coefficients (R) and correlation test *p*-values are given and a correlation line is plotted. Numeric values correspond to the five-point Likert scales centring the indiscriminate answers in middle where the grid line is plotted.

## Data Availability

The datasets used and/or analysed during the current study are available from the corresponding author on reasonable request.

## Abbreviation

ET: Eignungstest (the competitive entrance exam for Swiss medical schools)
