# Supplementary material for: Attractiveness of medical disciplines amongst Swiss first-year medical students allocated to different medical education tracks: cross-sectional study
Source: BMC Med Educ. 2022 Apr 7;22:252. doi: 10.1186/s12909-022-03313-x (PMC8986963; doi:10.1186/s12909-022-03313-x)
Supplement: Supplementary file 1 — Additional file 1: Supplementary file 1. Sankey plot showing proportions and (re-)assignment flow of applicants to specific medical education tracks in 2019. The first vertical bar from the left represents the total of applicants, the stack of vertical bars represents the proportion of applicants to specific medical education tracks, the third stack of bars the proportion of applicants passing the aptitude test (“Eignungstest”) and the forth stack of bars the actual allocation to specific medical education tracks. At each node the total number applicants is given. [file 12909_2022_3313_MOESM1_ESM.pdf]

Supplementary file 1

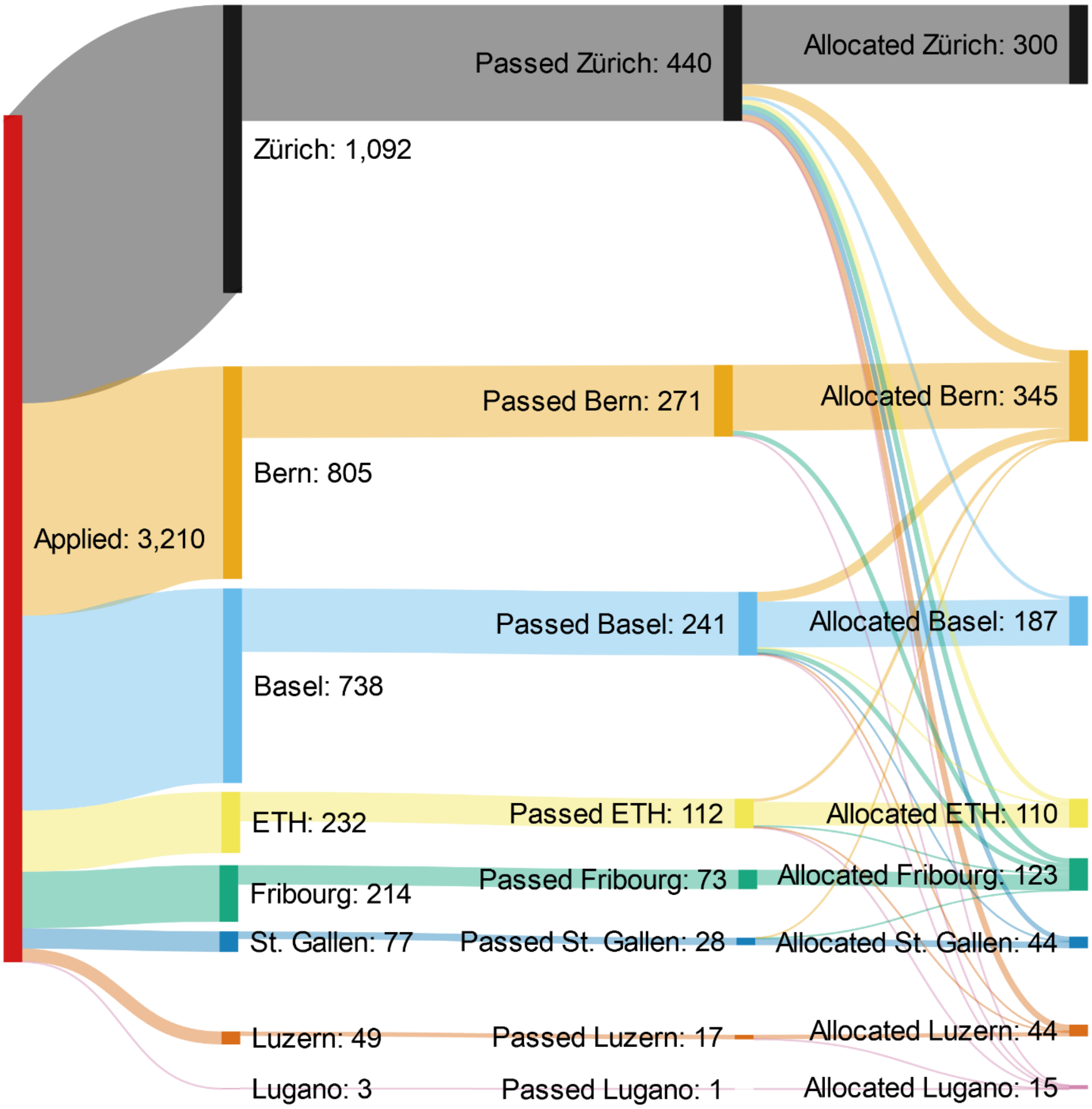

Sankey plot showing proportions and (re-)assignment flow of applicants to specific medical education tracks in 2019. The first vertical bar from the left represents the total of applicants, the stack of vertical bars represents the proportion of applicants to specific medical education tracks, the third stack of bars the proportion of applicants passing the aptitude test ("Eignungstest") and the forth stack of bars the actual allocation to specific medical education tracks. At each node the total number applicants is given
