# Supplementary material for: Attractiveness of medical disciplines amongst Swiss first-year medical students allocated to different medical education tracks: cross-sectional study
Source: BMC Med Educ. 2022 Apr 7;22:252. doi: 10.1186/s12909-022-03313-x (PMC8986963; doi:10.1186/s12909-022-03313-x)
Supplement: Supplementary file 2 — Additional file 2: Supplementary file 2. Questionnaire translated in English. [file 12909_2022_3313_MOESM2_ESM.pdf]

Survey of career goals and importance of career relevant determinants among Swiss medical students

Thank you very much for completing this survey (it will require not more than 5 minutes of your time). This study is currently executed on multiple Swiss universities simultaneously, your participation is completely voluntary. The results of this study will help to devellop future curricula please answer the questions according to your best konwledge. This survey is anonymous and no individial responses, only pooled results will be disseminated.

* 1. To which University did you originally apply to (before the Eignungstest)?

- ◯ University of Zurich (domestic track)
- ◯ University of Zurich (lucern track)
- ◯ University of Zurich (St. Gallen track)
- ◯ ETH Zurich
- ◯ University of Basel (domestic track)
- ◯ University of Basel (USI track)
- ◯ University of Bern
- ◯ University of Fribourg
- ◯ Other (please specify)

* 2. To which University were you assigned?

- ◯ University of Zurich (domestic track)
- ◯ University of Zurich (lucern track)
- ◯ University of Zurich (St. Gallen track)
- ◯ ETH Zurich
- ◯ University of Basel (domestic track)
- ◯ University of Basel (USI track)
- ◯ University of Bern
- ◯ University of Fribourg
- ◯ Other (please specify)

* 3. Please rate the attrativeness of potential careers below

	Absolutely not attractive (excluded career goal)	Rather unattractive	Neutral / can't say	rather attractive	Totally attractive (the only career goal)
general practice	○	○	○	○	○
gynecology / pediatrics	○	○	○	○	○
outpatient subspecialty (e.g. cardiology, gastroenterology, rheumatology)	○	○	○	○	○
inpatient general internal medicine	○	○	○	○	○
inpatient subspecialty (including chirurgcial disciplines)	○	○	○	○	○
Research at an university and academic career	○	○	○	○	○
Research and development in the industrial sector	○	○	○	○	○

4. Please rate the following determinants according to importance for your of career choice

	Very unimportant	Rather unimportant	Neutral / can't say	Rather important	Very important
Financial success	◯	◯	◯	◯	◯
reputation	◯	◯	◯	◯	◯
political environment	◯	◯	◯	◯	◯
part time work possibilities	◯	◯	◯	◯	◯
relations to patients	◯	◯	◯	◯	◯
having medical tasks	◯	◯	◯	◯	◯
career opportunities	◯	◯	◯	◯	◯
autonomy at work	◯	◯	◯	◯	◯

5. Your residency canton when you applied to medical school

When applied from abroad, please specify country

6. Your sex

- female
- male

7. Your age in years

8. Your score at the Eignungstest

9. Anonymizing code (to combine your answers in a follow-up survey without unveiling identifying information)

Birthday (day only without month or year)

First name of your mother (only the first 3 letters)

Count of your siblings

That's all! Thank you very much for participating.
