# Supplementary material for: Attractiveness of medical disciplines amongst Swiss first-year medical students allocated to different medical education tracks: cross-sectional study
Source: BMC Med Educ. 2022 Apr 7;22:252. doi: 10.1186/s12909-022-03313-x (PMC8986963; doi:10.1186/s12909-022-03313-x)
Supplement: Supplementary file 3 — Additional file 3: Supplementary file 3. Scatterplot matrix for all pairs of attractiveness of career goals and importance of factors associated with career. Spearman correlation coefficients (R) and correlation test p-values are given and a correlation line is plotted. Numeric values correspond to the five-point Likert scales centring the indiscriminate answers in middle where the grid line is plotted. [file 12909_2022_3313_MOESM3_ESM.pdf]

Supplementary file 3

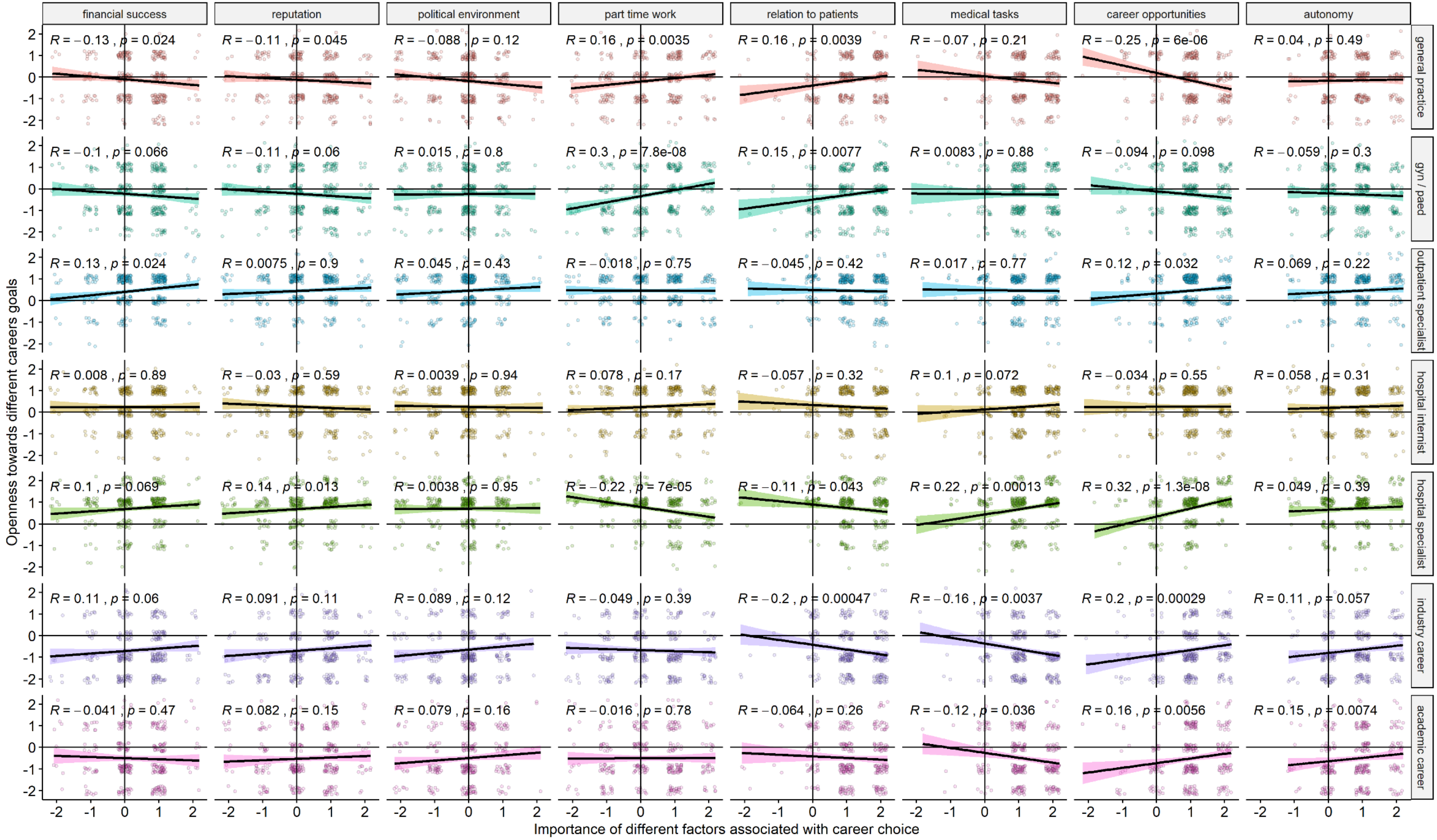

Scatterplot matrix for all pairs of attractiveness of career goals and importance of factors associated with career. Spearman correlation coefficients (R) and correlation test p-values are given and a correlation line is plotted. Numeric values correspond to the five-point Likert scales centring the indiscriminate answers in middle where the grid line is plotted
